# Cerebral hemorrhage due to amyloid angiopathy that was difficult to differentiate from breast cancer metastasis: a case report

**DOI:** 10.1186/s40792-021-01225-4

**Published:** 2021-06-27

**Authors:** Toshitaka Uomori, Yoshiya Horimoto, Masashi Takanashi, Ayana Shikanai, Katsuya Nakai, Astushi Arakawa, Mitsue Saito

**Affiliations:** 1 Department of Breast Oncology, Juntendo University School of Medicine, 2-1-1 Hongo, Bunkyo-ku, Tokyo, 113-0033 Japan; 2 Department of Human Pathology, Juntendo University School of Medicine, 2-1-1 Hongo, Bunkyo-ku, Tokyo, 113-0033 Japan; 3 Department of Neurology, Juntendo University School of Medicine, 2-1-1 Hongo, Bunkyo-ku, Tokyo, 113-0033 Japan

**Keywords:** Breast cancer, Brain metastasis, Cerebral amyloid angiopathy, Cerebral hemorrhage, Cognitive dysfunction

## Abstract

**Background:**

Breast cancer patients are known to develop brain metastasis at a relatively high frequency. However, imaging findings of brain metastases vary, and it is sometimes very difficult to distinguish these from other tumorous lesions and non-neoplastic lesions, such as cerebral hemorrhage. Meanwhile, there are various causes of cerebral hemorrhage; a major one is cerebral amyloid angiopathy (CAA). With the advancement of imaging technology, CAA-related cerebral hemorrhage can be more precisely diagnosed with magnetic resonance imaging (MRI), but definitive diagnosis of CAA can only be made based on pathological assessment. Herein, we report a case of consciousness disorder appearing during adjuvant therapy for breast cancer. We initially considered that the patient’s cerebral hemorrhage was due to a metastatic tumor, but based on excisional biopsy, she was diagnosed with CAA.

**Case presentation:**

A 73-year-old Japanese woman underwent curative surgery for left breast cancer. Her disease was hormone receptor-positive and human epidermal growth factor receptor 2 (HER2)-positive invasive ductal carcinoma (pStage IIB). While receiving adjuvant treatment, she developed disorientation, and emergent imaging revealed multiple cerebral hemorrhages. There was no apparent enhancement in the cerebral parenchyma on MRI, and differential diagnosis included hemorrhage due to a metastatic tumor, intravascular large B-cell lymphoma, CAA and thrombotic intracranial bleeding. After hospitalization, the bleeding lesion enlarged, resulting in cerebral hernia, and she needed emergency drainage surgery. The tissue surrounding the hemorrhage was pathologically assessed, and she was diagnosed with CAA. Although we initially suspected the lesion to be a metastatic tumor from breast cancer, there were no tumorous cells.

**Conclusion:**

Atypical MRI findings made diagnosis difficult in this case, but it should be considered for differential diagnosis when multiple cerebral hemorrhages in elderly patients are observed, especially in cases with symptoms such as transient multifocal neurological deficits and dementia.

## Background

Breast cancer patients develop brain metastasis at a relatively high frequency, along with lung cancer patients. Among breast cancer types, human epidermal growth factor receptor 2 (HER2)-positive tumors are more likely to develop brain metastases [1]. Meanwhile, the frequency of brain metastases found with symptoms is reportedly 10–16% [2], indicating that the majority of brain metastases are asymptomatic and have been found by imaging. Hence, accurate diagnostic imaging is crucial.

Breast cancer brain metastases are frequently observed in the subcortical white matter, but we have sometimes observed cerebellar and brain stem metastases. There are also metastases to the meninges and pachymeninx without forming mass lesions, thus diagnostic imaging findings vary [3, 4]. In contrast, tumorous lesions of the brain may be other neoplastic lesions, such as malignant lymphoma, which is sometimes difficult to distinguish. We recently reported a case in which a surgical biopsy was performed for a brain mass lesion that developed during treatment for Stage IV breast cancer. Even with pathological assessment, it was difficult to distinguish between metastatic breast cancer and diffuse large B-cell lymphoma [5]. Furthermore, it is also necessary to keep in mind the possibility of non-neoplastic lesions, such as cerebral hemorrhage.

There are various causes of cerebral hemorrhage, but one of the major causes of non-hypertensive cerebral hemorrhage, especially in the elderly, is cerebral amyloid angiopathy (CAA). In CAA, amyloid protein is deposited on the walls of small and medium-sized arteries, causing weakening of the blood vessels. It not only causes cerebral hemorrhage, but also cerebral infarction, leukoencephalopathy, and vasculitis. CAA can cause spontaneous cerebral hemorrhage, sometimes leading to sudden death, especially in the elderly. In a study on CAA-related cerebral hemorrhage in Japan, the average age of onset was 73.3 years and was more common in women, with a sex ratio of 2.2 [6]. In about 30% of cases, re-bleeding occurred with an average interval of 11.7 months. With the development of imaging technology, more CAA-related cerebral hemorrhage has been diagnosed by magnetic resonance imaging (MRI) with T2-weighted imaging (GRE method) or the SWI (susceptibility-weighted imaging) methods. Radiological features of CAA include multiple microbleeds on MRI T2* sequence. Although the Modified Boston criteria [7] are used clinically for CAA-related cerebral hemorrhage, pathological assessment is essential to reach definite diagnosis of CAA. Thus, it is not common to reach a definitive diagnosis. Nevertheless, CAA should be suspected in the presence of multiple subcortical hemorrhages in the absence of other possible causes.

Herein, we report a case in which consciousness disorder appeared during adjuvant therapy for breast cancer. We initially considered her disease to be a metastatic brain tumor, but reached a final diagnosis of bleeding due to CAA based on excisional biopsy during emergency drainage surgery.

## Case presentation

A 73-year-old Japanese woman was diagnosed with left breast cancer and underwent curative surgery at our institution. Final diagnosis was invasive ductal carcinoma, high-grade, hormone receptor-positive, HER2-positive, pT2N1aM0 (stage IIB). Regarding the patient’s medical history, she experienced hepatitis B and had adenomatous goiter. She also underwent surgery for meningioma in the right frontal lobe 30 years previously. For systemic adjuvant therapy for breast cancer, chemotherapy combined with anti-HER2 drugs was recommended, but the patient declined to receive cytotoxic chemotherapy. Moreover, a decrease in cognitive ability had been observed since the start of treatment for breast cancer. Considering the situation, only anti-HER2 treatment with endocrine therapy (aromatase inhibitor) was administered.

Two months before the emergent hospitalization, 4 months after the curative surgery for breast cancer, she visited an emergency outpatient service because she had lost the ability to use a kitchen knife and had developed urinary incontinence at night. However, there was no specific finding on the brain computed tomography (CT), and she was diagnosed with suspected symptomatic epilepsy. Thereafter, she experienced slow cognitive decline and was under examination on suspicion of dementia with Lewy bodies. One morning, she could not move her legs forward while standing. Emergency brain CT revealed multiple cerebral hemorrhages, and she was admitted to our hospital. There was no obvious quadriplegia, and no laterality was observed with a pupil of 3 mm in diameter. Blood tests showed no evidence of inflammation, and tumor markers were within normal limits. Adjuvant endocrine therapy and trastuzumab had been given until the hospitalization.

**Fig. 1:**
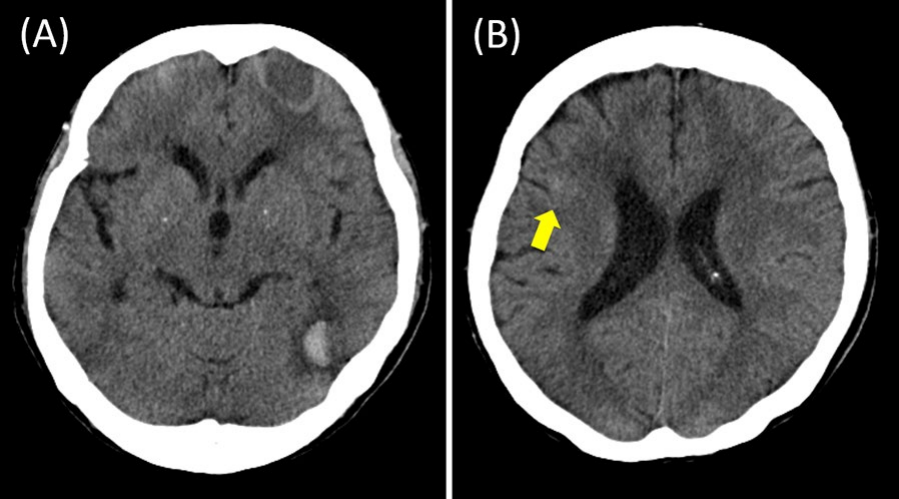
CT scan findings on the day of hospital admission. **A** A high-absorption area in the deep part near the posterior horn of the left lateral ventricle was found. The surrounding areas were accompanied by edema. A cystic lesion located in the anterior left frontal lobe indicates a postoperative change of meningioma. **B** A faint high-absorption area was observed in the sulcus near the right frontal operculum (yellow arrow)

**Fig. 2:**
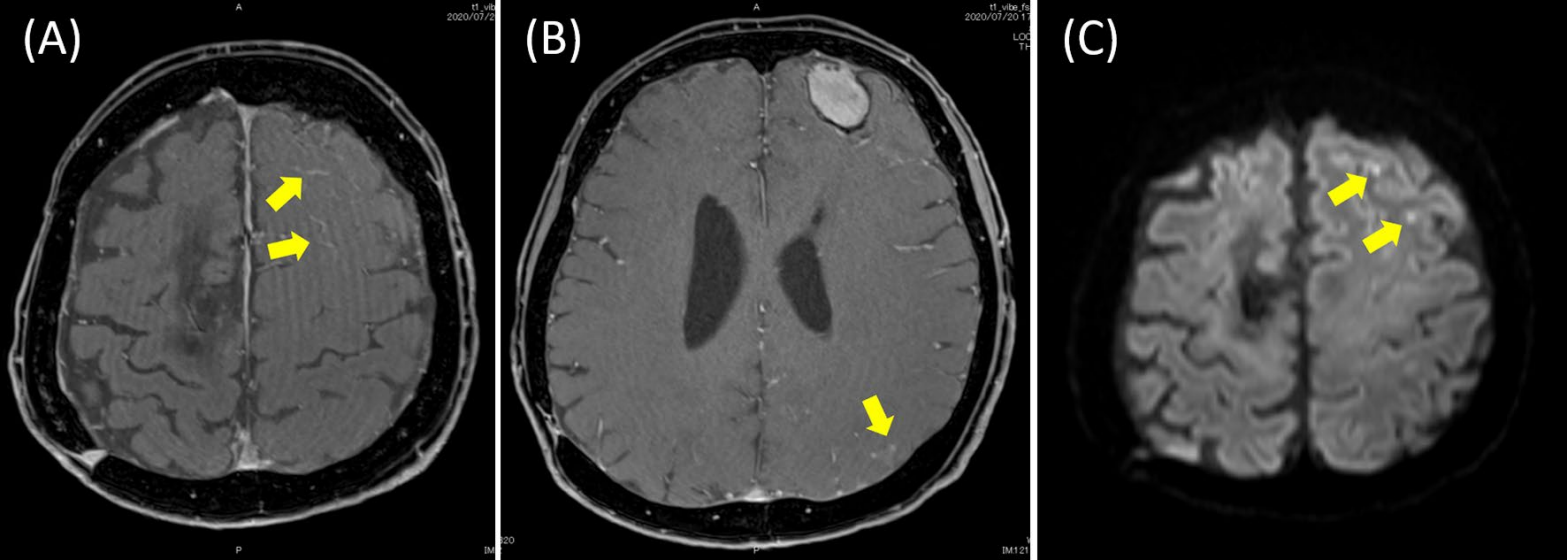
Contrast-enhanced MRI findings. **A** The T1-weighted image showed hyperintensity on the anterior left frontal lobe and lateral floor of the left temporal lobe (yellow arrows), with edema in the surrounding tissue. **B** The cerebral surface of the pia mater was slightly contrast-enhanced (yellow arrow), but there was no apparent enhancement in the cerebral parenchyma. A cystic lesion located in the anterior left frontal lobe indicates a postoperative change of meningioma as described in Fig. 1A. **C** A small number of minimum hemosiderin deposits (yellow arrows) were observed near the cortex, but no findings strongly suggestive of amyloid angiopathy were observed

**Fig. 3:**
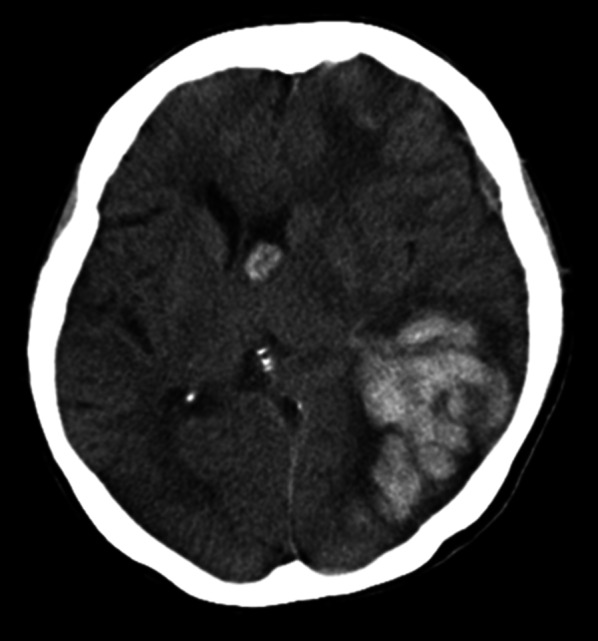
CT scan on the day when the patient developed consciousness disorder. Bleeding near the posterior horn of the left lateral ventricle was prominently enlarged to 8 cm in diameter, and left uncus herniation and subfalcian herniation were evident

On the CT scan, a high-absorption area indicating bleeding was found in the deep part near the posterior horn of the left lateral ventricle (Fig. 1). After hospital admission, the neurosurgical department was consulted, and conservative treatment using anti-hemorrhagic agents were given. On the next day, contrast-enhanced MRI was conducted, and T1-weighted imaging showed hyperintensity on the anterior left frontal lobe and lateral floor of the left temporal lobe, with edema in the surrounding tissue (Fig. 2). The T2-weighted image produced a lower signal, thus these lesions were suspected to be cerebral hemorrhages. The cerebral surface of the pia mater was slightly contrast-enhanced, but there was no apparent enhancement in the cerebral parenchyma. Differential diagnosis included hemorrhage due to metastatic tumor, intravascular large B-cell lymphoma, CAA and thrombotic intracranial bleeding. The next day, her consciousness level dropped dramatically. A CT scan revealed the bleeding near the posterior horn of the left lateral ventricle had enlarged to 8 cm in diameter (Fig. 3). She developed left uncal and subfalcine herniation and emergency drainage surgery was conducted.

**Fig. 4:**
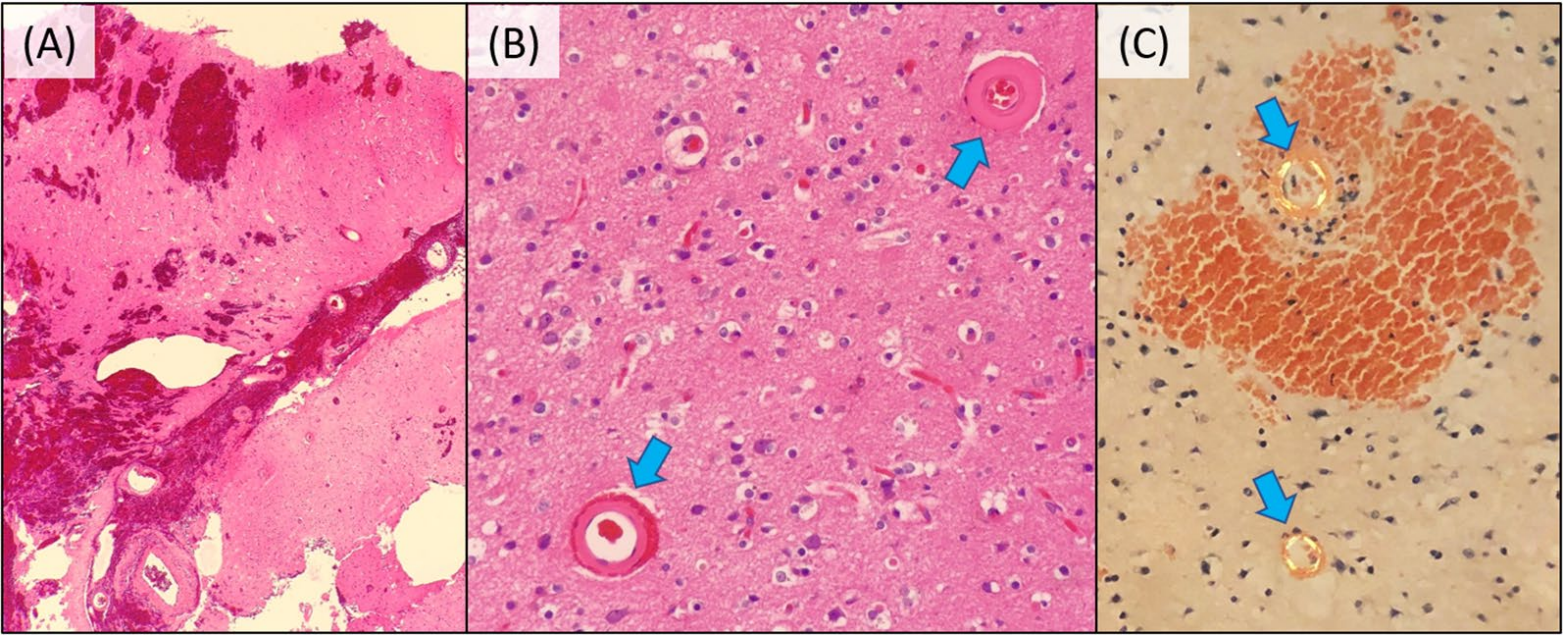
Pathological features of the tissue surrounding the hemorrhage. **A** Hematoxylin and eosin (HE) staining: brain tissue surrounding the hemorrhage (× 40). **B** HE staining: walls of small arteries (blue arrows) were thickened (× 200). **C** Congo-Red staining: green birefringence was observed under polarizing microscope (blue arrows), indicating amyloid deposition (× 200)

Figure 4 indicates the pathological features of the tissue surrounding the hemorrhage obtained intraoperatively. With gliosis in the background brain tissue, there were small arteries with thickened walls, and with amyloid deposits in the walls. No tumorous cells were found, although we had initially suspected the lesion to be a metastatic tumor from breast cancer. Therefore, she was pathologically diagnosed with CAA.

The patient was then transferred to another hospital for rehabilitation. After transfer, she developed re-bleeding and has been bedridden requiring total assistance.

## Conclusion

The patient’s episodes were typical clinical predictors for CAA, but the MRI finding without multiple microbleeds on T2* sequence was atypical. This discrepancy made for a complicated diagnosis, even for neurosurgeons. Aging is the highest risk factor for CAA. Therefore, when an elderly patient develops transient multifocal neurological deficits and dementia, CAA should be considered in the differential diagnosis. We hope our report will allow clinicians to make a broader differential diagnosis in similar situations.

## Abbreviations

CAA: Cerebral amyloid angiopathy
CT: Computed tomography
HER2: Human epidermal growth factor receptor 2
MRI: Magnetic resonance imaging
